# Follicular dynamics predict optimal insemination timing in Yorkshire, Duroc, and Landrace × Yorkshire gilts

**DOI:** 10.3389/fvets.2026.1946137

**Published:** 2026-09-22

**Authors:** Zhicheng Shi, Lichun He, Shanli Zhu, Xin Zhou, Qing Zhang, Wenchao Li, Junrong Li

**Affiliations:** 1 College of Agriculture, Jinhua University of Vocational Technology, Jinhua, China; 2 Jinhua Key Laboratory of Genetic Breeding and Improvement on Jinhua Pig, Jinhua, China; 3 College of Animal Science and Technology, China Agricultural University, Beijing, China; 4 Changnong, Da Bei Nong Agriculture, Animal Husbandry and Food Co., Ltd, Wenshan Zhuang and Miao Autonomous Prefecture, Yunnan, China

**Keywords:** artificial insemination, estrus-to-ovulation interval, follicular development, gilts, ovarian ultrasound

## Abstract

Accurate characterization of estrus and ovulation is critical for effective artificial insemination (AI) in pigs, yet comparative data on ovulatory dynamics among Yorkshire, Duroc, and Landrace × Yorkshire gilts remain scarce. In this study, transabdominal ultrasound was used to monitor ovarian activity in these three genetic lines following altrenogest-based estrus synchronization. For each genetic line, gilts were monitored at 8-h intervals. Landrace × Yorkshire crossbred gilts exhibited a significantly shorter altrenogest-withdrawal-to-ovulation interval (*P* < 0.05). In the full cohorts (Yorkshire, *n* = 200; Duroc, *n* = 90; Landrace × Yorkshire, *n* = 504), Yorkshire gilts had the highest litter size, whereas Duroc gilts developed larger pre-ovulatory follicles (based on a subset of 17 gilts per breed) but had lower litter size. A negative correlation between estrus-onset timing and the estrus-to-ovulation interval was observed across all genetic lines. Farrowing rates exceeded 90% in all groups. These findings provide critical physiological data for designing breed-specific precision breeding strategies in commercial pig production.

## 1 Introduction

Yorkshire, Duroc, and Landrace are globally recognized as foundational pig breeds and are widely used as parental lines due to their superior genetic merit. Crossbred combinations, notably Landrace × Yorkshire, have been developed to synergistically combine the favorable traits of these parent breeds (1, 2). In contrast to fur-bearing species (e.g., foxes, raccoon dogs, and minks), where production emphasis lies primarily on fur quality, pig production prioritizes reproductive performance and carcass yield (3).

In recent years, shifting consumer preferences toward leaner, higher-quality pork have driven the demand for more efficient breeding strategies. Consequently, Landrace × Yorkshire has become a dominant maternal line in China's commercial pig industry, valued for its excellent reproductive efficiency and adaptability to intensive farming systems (4). Fixed-time artificial insemination (FTAI), which relies on hormonal synchronization to enable insemination at a predetermined time without estrus detection, has facilitated “all-in-all-out” production systems. However, FTAI continues to be hindered by reproductive inefficiencies (5, 6). Exogenous gonadotropins such as eCG can stimulate ovulation, but their use is associated with compromised embryonic viability and reduced pregnancy rates (6, 7). High frequencies of implantation failure and embryonic loss substantially reduce litter size, representing a major biological constraint that has limited the commercial application of FTAI for decades (8, 9). These challenges underscore the need for refined reproductive protocols that minimize reliance on exogenous hormones while maintaining synchronization efficiency.

The timing of insemination remains a critical determinant of reproductive performance (10, 11). Under natural conditions, the estrus-to-ovulation interval is both prolonged and highly variable, ranging from approximately 33.0–99.0 h, with a reported mean of 63.6 h (12). In contrast, hormonal synchronization protocols effectively narrow the ovulation window, typically occurring 30–40 h post-estrus (13). Thus, elucidating breed-specific variation in reproductive physiology—particularly among Yorkshire, Landrace, and Duroc gilts—is essential for designing precise breeding strategies, especially in multi-breed production systems. Notably, direct comparative data on ovulatory characteristics across these three major breeds remain scarce. Therefore, the present study aimed to characterize breed-specific ovulatory dynamics in these three genetic lines using serial transabdominal ultrasound, and to evaluate whether ultrasonographic parameters could guide individualized AI timing.

## 2 Materials and methods

This experiment was carried out in cooperation with Yunnan Changnong Agriculture and Animal Husbandry Food Co., Ltd. (Yanshan County, Wenshan Zhuang and Miao Autonomous Prefecture, Yunnan, China). The animal protocol was approved by the Institutional Animal Care and Use Committee of China Agricultural University (No. AW80804202-1-1).

### 2.1 Animals and experimental design

To investigate breed-specific differences in follicular development and ovulation, Yorkshire (*n* = 200), Landrace × Yorkshire (*n* = 504), and Duroc (*n* = 90) gilts were used, with age differences ≤ 3 days across the three genetic lines. Selected gilts (17gilts of each breeds) were individually monitored, and the first two estrous cycles were recorded before altrenogest treatment (~8 months). Back fat thickness was measured at the beginning of experiment using an Exgo.s ultrasound device with the following settings: Gain, 80 dB; Depth, 90 mm; ENH, 1; DR, 60 and Frequency, 5.0 MHz. Back fat thickness was restricted to 14–18 mm for enrolled animals. All gilts were individually housed in gestation stalls (2.2 m × 0.6 m; 1.32 m^2^ per gilt) on fully slatted floors (slat openings: 20 mm). Gilts were individually housed under controlled conditions (22 ± 1 °C, 60%−70% humidity, 16-hour light/8-h dark).

For estrus synchronization, 3 days before altrenogest administration, gilts in both groups received 5 mL of glucose syrup via a feeding gun to acclimatize them to the feeding procedure. Thereafter, altrenogest (Ningbo Sansheng, Zhejiang, China) was orally administered at a dose of 20 mg per head daily at 15:00 for 18 consecutive days. From the day after the last altrenogest administration, estrus detection and ultrasonographic monitoring commenced, using a portable HS-1,600V veterinary ultrasound system (Honda Electronics, Japan). AI was performed 8–12 h after estrus onset, with a second insemination 24 h later. The experimental timeline is illustrated in Figure 1.

**Figure 1:**
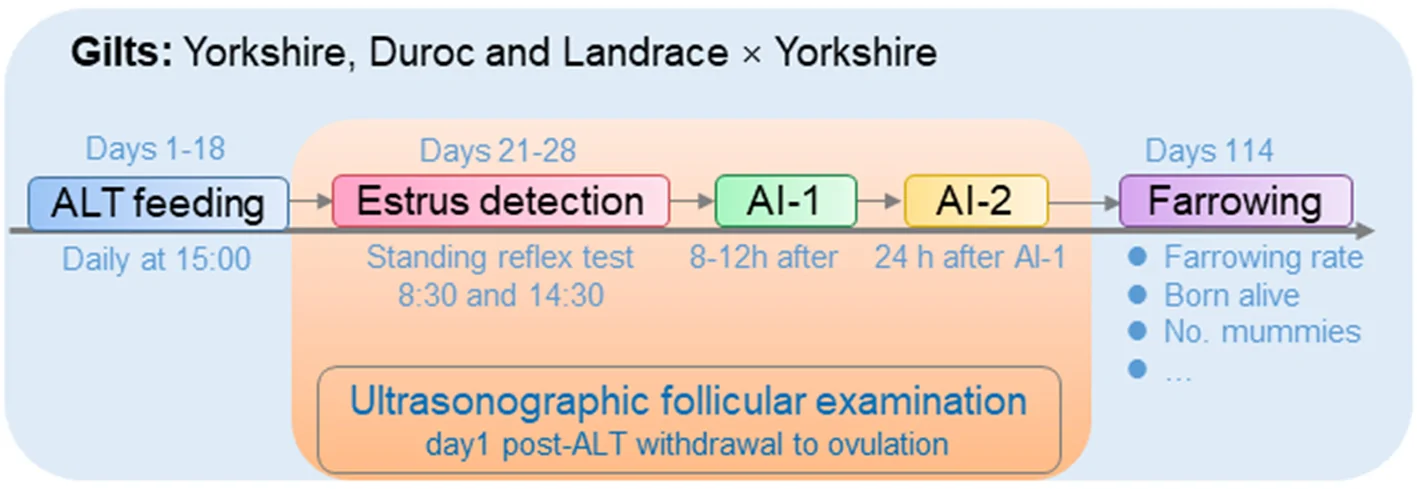
Schematic illustration of the experimental design of the Yorkshire, Duroc and Landrace × Yorkshire breeds. ALT: altrenogest.

### 2.2 Estrus detection and ovulation monitoring

Estrus detection was performed twice daily (08:30 and 14:30), starting 3 days after the last altrenogest administration. Each gilt was exposed to a mature, sexually active boar and the standing reflex was recorded. The onset of estrus was defined as the first clear standing response.

Transabdominal ultrasound was performed at 8-h intervals (00:30, 08:30, and 16:30) from 1 day after altrenogest withdrawal until ovulation was confirmed. Briefly, for each scan, the probe was placed over the penultimate mammary gland, and gentle transabdominal pressure was applied. Real-time B-mode imaging was performed by fan-sweeping the probe along the inner thigh, with the probe angle adjusted to optimize visualization of follicles (14). The diameter of the largest follicle was recorded daily to monitor growth; during estrus, pre-ovulatory follicles typically exceeded 5 mm. To ensure accurate assessment of follicle diameter, three visible follicles were measured on each ovary, and the mean diameter was calculated and recorded as the follicle size for that time point. Ovulation was determined by sequential follicular disappearance, using the following criteria: (1) if only one or two dominant follicles (>5 mm) remained at a given scan, with subordinate follicles < 3 mm, ovulation was assigned to that time point; (2) if complete collapse of all large follicles occurred between two consecutive scans, ovulation was considered to have taken place within that 8-h interval. For each gilt, follicular diameter at estrus onset and the estrus to ovulation interval were subsequently calculated for each gilt.

### 2.3 Artificial insemination and reproductive performance assessment

Semen was collected according to standard procedures (15). Each insemination dose contained 60 mL of extended semen with a total of 1.5–2.0 × 10^9^ spermatozoa, with motility ranging from 77.0 to 85.0%. AI was performed 8–12 h after estrus onset, with a second insemination 24 h later. Each gilt received 2 semen doses in total. For each gilt, the second AI dose was derived from the same boar and the same ejaculate batch as the first dose, ensuring that all inseminations for a given gilt were performed with homospermic semen from a single boar. Landrace × Yorkshire gilts received semen of Yorkshire to maintain a consistent terminal sire effect across maternal lines. For all farrowed pigs, we recorded farrowing rate, total piglets born, live-born piglets, stillbirths, mummified fetuses and litter weight.

### 2.4 Statistical analysis

Data were analyzed using one-way ANOVA with breed as fixed effect, after confirming normality (Shapiro–Wilk) and homogeneity of variances (Levene's test). For reproductive traits (backfat thickness, weaning-to-estrus interval, farrowing performance), full-cohort data (Yorkshire *n* = 200, Duroc *n* = 90, Landrace × Yorkshire *n* = 504) were subjected to Tukey–Kramer adjustment for multiple comparisons due to unequal group sizes. For ovulation-related traits (intervals from altrenogest withdrawal and estrus to ovulation), assessed via ultrasonography in 17 gilts per genetic line (balanced design), Tukey's HSD test was used. Results are presented as mean ± SEM, with statistical significance set at *P* < 0.05. Exact sample sizes for each trait are specified in the corresponding table/figure legends.

## 3 Results

### 3.1 Estrus response following altrenogest withdrawal

Mean backfat thickness was 14.36, 14.58, and 15.11 mm for the three genetic lines, respectively (*P* > 0.05). After altrenogest withdrawal, most gilts showed estrus on Days 6–7 (59.63%, 63.08%, and 62.34%, respectively), and cumulative estrus rates were 92.13%, 97.02%, and 93.80% (*P* > 0.05).

### 3.2 Follicular development and ovulation timing

Following the withdrawal of altrenogest, follicular development was monitored, especially at estrus and ovulation. Typical ultrasound images are displayed in Figure 2A. Landrace × Yorkshire gilts exhibited significantly shorter interval from withdrawal to ovulation than the other two breeds (Table 1, *P* < 0.05). Moreover, the changes in follicle diameter over this period are shown in Table 1. Although follicle diameter at estrus onset was similar across breeds (*P* > 0.05), Duroc gilts developed significantly larger follicles than Yorkshire and Landrace × Yorkshire gilts at 6.5, 7, and 7.5 days after altrenogest withdrawal (Figure 2B; *P* < 0.05 for each time point).

**Figure 2:**
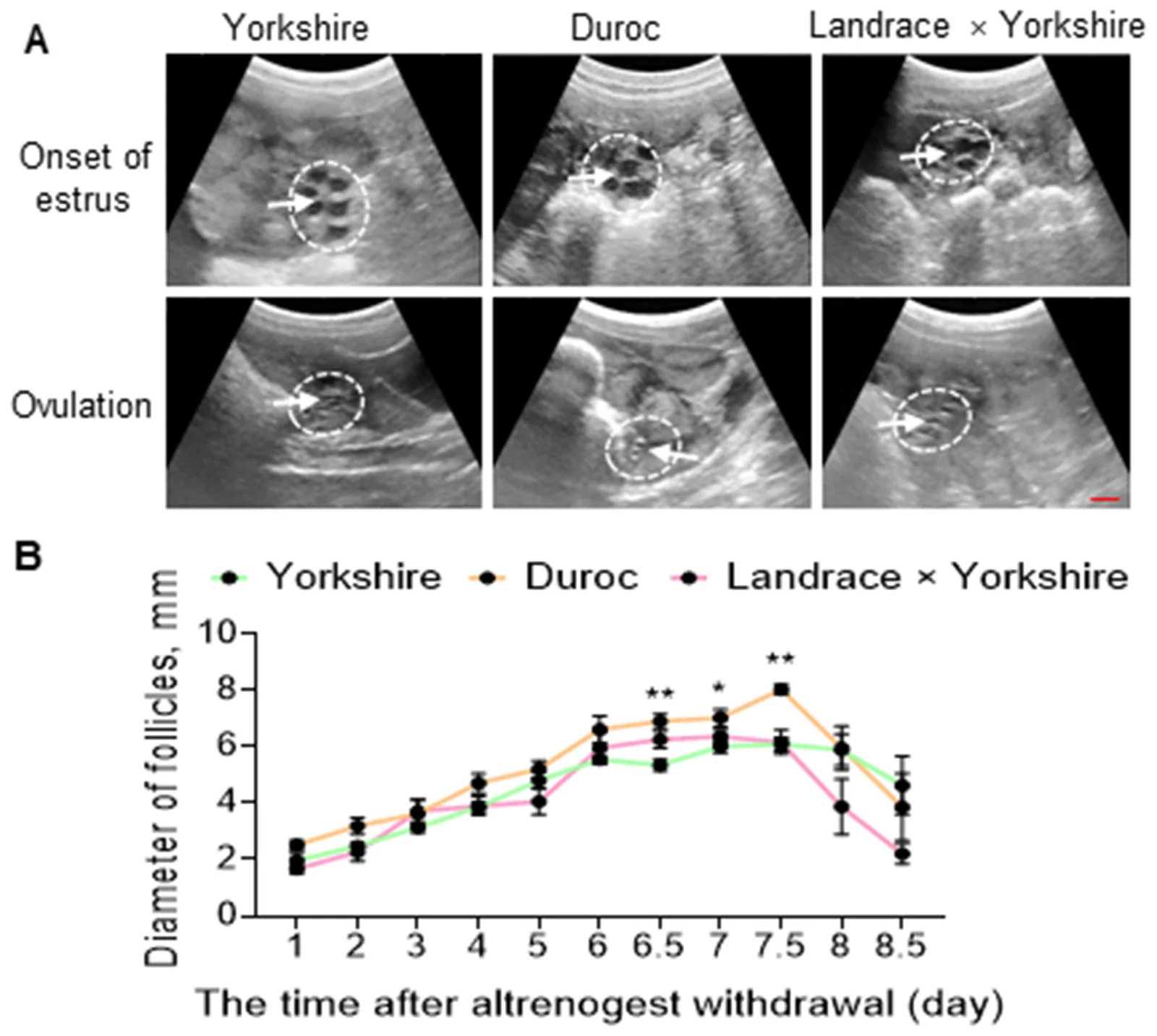
Ovulation characteristics in Yorkshire, Duroc, and Landrace × Yorkshire gilts. **(A)** Representative images of estrus and ovulation, bar is 10 μm. **(B)** Follicle diameter changes from altrenogest withdrawal to ovulation. Data are presented as mean ± SEM. Different letters indicate significant differences (*P* < 0.05). *, *P* < 0.05; **, *P* < 0.01. For ultrasonographic monitoring, a subset of 17 animals per genetic line was used.

**Table 1:** Altrenogest withdrawal-to-estrus interval and litter performance in different strains of gilts.

Item	Yorkshire	Duroc	Landrace × Yorkshire	*P* value
AWO interval (h)	159.27 ± 3.59^a^	152.80 ± 3.74^a^	134.78 ± 3.13^b^	< 0.0001
AWE interval (h)	129.00 ± 3.44	128.29 ± 4.54	117.41 ± 2.83	0.198
EOI interval (h)	35.07 ± 3.06	37.62 ± 2.93	32.42 ± 2.46	0.850
Farrowing rate (%)	93.33 ± 2.03	92.77 ± 1.89	93.40 ± 1.06	0.9600
Total piglets born (*n*)	15.11 ± 0.37^a^	9.60 ± 0.21^c^	12.45 ± 0.36^b^	< 0.0001
Born alive (*n*)	13.37 ± 0.34^a^	9.33 ± 0.34^c^	10.97 ± 0.20^b^	< 0.0001
No. stillbirth (%)	0.09 ± 0.01^a^	0.10 ± 0.02^a^	0.05 ± 0.01^b^	< 0.0001
No. mummies (%)	0.03 ± 0.01^b^	0.07 ± 0.02^a^	0.03 ± 0.01^b^	< 0.0001
Total litter weight (kg)	17.28 ± 0.43^a^	11.90 ± 0.25^b^	12.49 ± 0.43^b^	< 0.0001
Average birth weight (kg)	1.31 ± 0.02^b^	1.46 ± 0.04^a^	1.21 ± 0.01^c^	< 0.0001

Results are presented as mean ± SEM, different superscript letters in the same row indicate significant differences (*P* < 0.05). AWO, altrenogest withdrawal to ovulation; AWE, altrenogest withdrawal to estrus; EOI, estrus to ovulation, *n* = 17. The sample sizes for the evaluated litter performance traits were as follows: 200 for Yorkshire, 504 for Landrace × Yorkshire, and 90 for Duroc. ^a, b, c^ Indicate statistically significant differences among the groups.

### 3.3 Correlation between estrus-to-ovulation interval and estrus onset time

Pearson correlation analysis confirmed a significant negative relationship between the withdrawal-to-estrus interval and the estrus-to-ovulation interval in Yorkshire, Duroc, and Landrace × Yorkshire gilts (*P* < 0.01).

### 3.4 Breed-specific reproductive performance

Farrowing rates exceeded 90% in all three genetic groups. Yorkshire gilts outperformed the other two genetic groups in total litter size, born alive, and total litter weight (*P* < 0.05). Duroc gilts exhibited the lowest total piglets born and born alive (*P* < 0.05), accompanied by a higher incidence of mummified fetuses (*P* < 0.05) and greater average birth weight (*P* < 0.05). Landrace × Yorkshire gilts displayed intermediate values for most traits (Table 1).

## 4 Discussion

The precise temporal alignment of estrus and ovulation is essential to reproductive efficiency in pigs, yet comparative data on ovulatory dynamics among Yorkshire, Duroc, and Landrace × Yorkshire gilts remain limited (16). In this study, we systematically characterized follicular development and ovulation patterns in these three genetic lines using serial transabdominal ultrasound following altrenogest synchronization. Our results reveal that while the overall estrus and ovulation responses are largely conserved across genotypes, distinct breed-specific characteristics exist that directly inform AI scheduling.

First, a breed-specific difference was observed in the altrenogest-withdrawal-to-ovulation interval. Landrace × Yorkshire gilts exhibited a significantly shorter interval from withdrawal to ovulation compared with purebred counterparts, whereas the intervals from withdrawal to estrus onset and from estrus onset to ovulation did not differ among groups. This indicates that the shortened window in crossbred gilts is primarily driven by earlier estrus onset after altrenogest withdrawal rather than a compressed estrus-to-ovulation interval *per se*, reflecting a heterosis effect.

Second, Duroc gilts developed significantly larger pre-ovulatory follicles during the post-withdrawal period (Figure 2B), despite comparable follicle diameters at estrus onset across breeds. This enlarged follicular size may reflect breed-specific differences in gonadotropin responsiveness and could influence oocyte developmental competence, warranting further investigation. The reduced litter size and increased birth weight observed in Duroc gilts may reflect a trade-off between total piglets born and intrauterine growth, potentially linked to the larger follicular size observed in this genetic line.

Third, we identified a significant negative correlation between the altrenogest-withdrawal-to-estrus interval and the estrus-to-ovulation interval across all three breeds (Figures 3A–C). This finding enables more precise, individualized AI scheduling without additional hormonal intervention. In practical terms, gilts that express estrus later in the synchronized period may benefit from earlier insemination relative to the standard protocol, while those with earlier estrus onset may tolerate a slightly delayed insemination without compromising fertilization. Mechanistically, this correlation aligns with the established temporal association between the luteinizing hormone (LH) surge and estrus onset: under natural conditions, the LH peak consistently appears within 12 h after estrus (17). In gonadorelin-treated gilts, the interval from estrus onset to the LH peak is approximately 3 h (18). In our parallel studies, the extended interval observed in buserelin-treated groups may better support cytoplasmic maturation of the oocyte and quality maintenance under induced ovulation conditions, potentially enhancing oocyte developmental competence (19–21).

**Figure 3:**
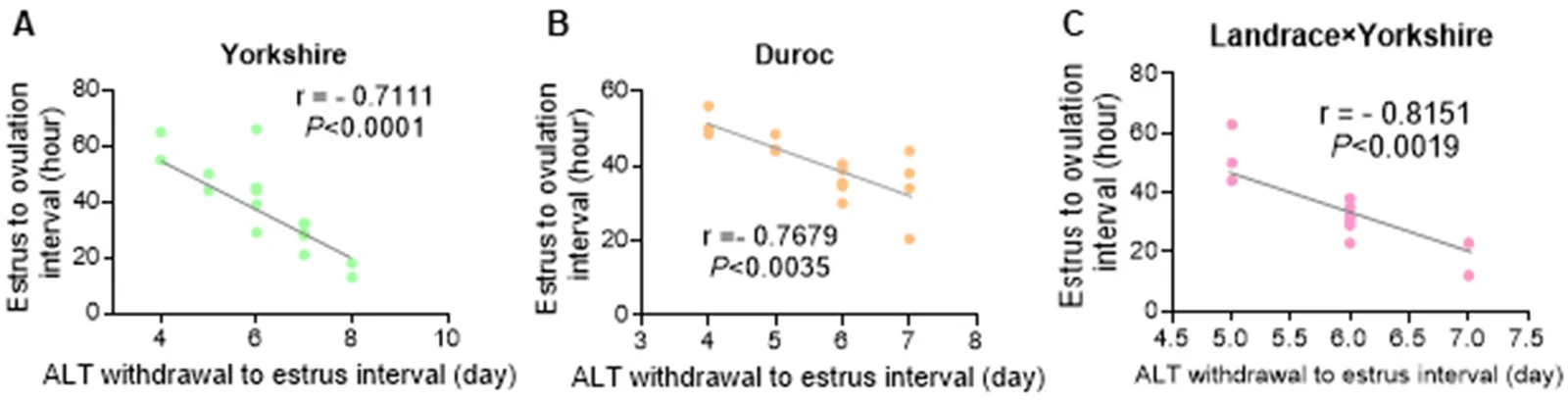
Correlation between altrenogest-withdrawal-to-estrus interval and estrus-to-ovulation interval in gilt strains. **(A)** Yorkshire, **(B)** Duroc and **(C)** Landrace × Yorkshire gilts. ALT: altrenogest. A sample size of 17 animals was used in each group.

The biological relevance of this refined timing is underscored by the established principle that fertilization rates and embryo counts peak when insemination occurs within 0–12 h before ovulation, whereas a 24-h delay post-ovulation reduces fertilization efficiency to below 40% (22). This temporal precision is particularly critical in multi-breed production systems, where synchronized estrus may mask underlying breed-specific variation in ovulatory timing. The ultrasonographic approach described here offers a practical, non-invasive means to refine AI timing without the cost and potential side effects of hormonal induction. By applying the present artificial insemination protocol based on ultrasonographically defined ovulatory dynamics, we achieved farrowing rates exceeding 90% across all genetic groups, validating the physiological appropriateness of our timing regimen. However, we acknowledge a limitation in the present study: we did not account for the boar semen effect during insemination, although a previous study based on 11,654 farrowing records reported that semen from different sources had no significant effects on total born or live born in sows of the same breed (23).

Collectively, our findings demonstrate that real-time follicular monitoring not only clarifies breed-specific ovulatory patterns but also supports the design of precise, genotype-adapted AI protocols. This approach offers a viable route to enhancing reproductive efficiency in multi-breed production systems while avoiding the challenges associated with gonadotropin-based synchronization, ultimately contributing to sustainable high-quality pork production.

## 5 Conclusion

Ultrasonographic monitoring in Yorkshire, Duroc, and Landrace × Yorkshire gilts revealed conserved estrus patterns but breed-specific ovulatory traits, including a shorter ovulation window in crossbreds and larger follicles in Durocs. The negative correlation between estrus onset and the estrus-to-ovulation interval enabled individualized AI timing without exogenous hormones, achieving > 90% farrowing rates and supporting a genotype-adapted reproductive management strategy.

## Data Availability

The original contributions presented in the study are included in the article/supplementary material, further inquiries can be directed to the corresponding author.
